# A pre-vertebrate endodermal origin of calcitonin-producing neuroendocrine cells

**DOI:** 10.1242/dev.202821

**Published:** 2024-08-07

**Authors:** Jenaid M. Rees, Katie Kirk, Giacomo Gattoni, Dorit Hockman, Victoria A. Sleight, Dylan J. Ritter, Èlia Benito-Gutierrez, Ela W. Knapik, J. Gage Crump, Peter Fabian, J. Andrew Gillis

**Affiliations:** ^1^ Department of Zoology, University of Cambridge, Cambridge CB2 3EJ, UK; ^2^ Department of Biological Sciences, Columbia University, New York City, NY 10027, USA; ^3^ Division of Cell Biology, Department of Human Biology, University of Cape Town, Cape Town 7935, South Africa; ^4^ Neuroscience Institute, University of Cape Town, Cape Town 7935, South Africa; ^5^ Department of Cell and Developmental Biology, Vanderbilt School of Medicine, Nashville, TN 37240, USA; ^6^ Vanderbilt Genetics Institute, Vanderbilt School of Medicine, Nashville, TN 37232, USA; ^7^ Eli and Edythe Broad Center for Regenerative Medicine, Department of Stem Cell Biology and Regenerative Medicine, Keck School of Medicine, University of Southern California, Los Angeles, CA 90033, USA; ^8^ Josephine Bay Paul Center for Comparative Molecular Biology and Evolution, Marine Biological Laboratory, Woods Hole, MA 02543, USA

**Keywords:** Calcitonin, Endoderm, Evolution, Neural crest, Neuroendocrine

## Abstract

Vertebrate calcitonin-producing cells (C-cells) are neuroendocrine cells that secrete the small peptide hormone calcitonin in response to elevated blood calcium levels. Whereas mouse C-cells reside within the thyroid gland and derive from pharyngeal endoderm, avian C-cells are located within ultimobranchial glands and have been reported to derive from the neural crest. We use a comparative cell lineage tracing approach in a range of vertebrate model systems to resolve the ancestral embryonic origin of vertebrate C-cells. We find, contrary to previous studies, that chick C-cells derive from pharyngeal endoderm, with neural crest-derived cells instead contributing to connective tissue intimately associated with C-cells in the ultimobranchial gland. This endodermal origin of C-cells is conserved in a ray-finned bony fish (zebrafish) and a cartilaginous fish (the little skate, *Leucoraja erinacea*). Furthermore, we discover putative C-cell homologs within the endodermally-derived pharyngeal epithelium of the ascidian *Ciona intestinalis* and the amphioxus *Branchiostoma lanceolatum*, two invertebrate chordates that lack neural crest cells. Our findings point to a conserved endodermal origin of C-cells across vertebrates and to a pre-vertebrate origin of this cell type along the chordate stem.

## Teaser

**Summary:** Vertebrate calcitonin-producing neuroendocrine cells derive from endoderm, not from neural crest as previously thought, and evolved from an ancient pharyngeal cell type of invertebrate chordates.

## INTRODUCTION

Calcitonin-producing neuroendocrine cells (‘C-cells’) are a specialized cell type of vertebrate animals that secretes the small peptide hormone calcitonin in response to elevated blood calcium (hypercalcaemia) (Copp and Cheney, 1962; Copp et al., 1962; Pearse, 1966a,b). In bony vertebrates, macrophage-like cells called osteoclasts resorb and remodel bone, releasing calcium into the bloodstream. Calcitonin from C-cells, in turn, lowers blood calcium levels by inhibiting osteoclast activity (Chambers and Moore, 1983; Nicholson et al., 1986) and promoting calcium deposition within bone (Talmage et al., 1980). Calcitonin is widely used for the acute treatment of metabolic bone disorders, such as osteoporosis (Muñoz-Torres et al., 2004) and Paget's disease (Langston and Ralston, 2004) – though, paradoxically, humans with varying levels of endogenous calcitonin [e.g. persons that have undergone thyroidectomy or with C-cell-derived medullary thyroid carcinoma (MTC)] exhibit no differences in bone mineral density (Hurley et al., 1987; Wuster et al., 1992). Nevertheless, osteoclast function is highly sensitive to calcitonin levels *in vitro* (Zaidi et al., 1987), and a mouse model of calcitonin deficiency (i.e. the *Calca*^−/−^ mouse, which lacks the gene encoding both calcitonin and its splice variant, calcitonin gene-related peptide) exhibits increased bone resorption with aging and decreased bone mineral density during lactation (Hoff et al., 2002; Janine et al., 2006). In cartilaginous fishes (sharks, skates and rays), calcitonin is reported to decrease blood calcium levels in some taxa (Srivastav et al., 1998) and increase blood calcium in others (Glowacki et al., 1985). Cartilaginous fishes lack bone and osteoclasts but, in these fishes, calcitonin may act on the gallbladder to regulate calcium concentrations and excretion in bile (Suzuki et al., 2009). Calcitonin therefore appears to function as a conserved modulator of calcium homeostasis in vertebrates – and possibly more broadly among animals (Cardoso et al., 2020) – though its mechanism(s) of action and responsive cell types remain incompletely understood outside of mammals (Dean et al., 2015).

In jawed vertebrates, C-cells develop within the ultimobranchial bodies (UBs): paired structures that derive from caudal pharyngeal pouches during early craniofacial development (Hamilton et al., 1945). Mammalian UBs are transient structures that develop from the fourth pharyngeal pouches and that ultimately migrate toward the midline to merge with the developing thyroid primordium (Nilsson and Fagman, 2017). This accounts for the final location of mammalian C-cells (also known as parafollicular cells) within the thyroid gland. In non-mammalian vertebrates (i.e. birds, reptiles, amphibians and fishes), UBs do not fuse with the thyroid gland and instead persist as distinct, paired C-cell-containing glands in the neck or caudal pharynx (Tauber, 1967; Copp et al., 1967; Kameda, 2017).

Though the location of C-cells within the UB (or mammalian thyroid gland) is well established, the embryonic origin of the cell type has been a matter of debate for over 50 years. Pearse, Polak, LeDouarin and colleagues reported a neural crest origin of avian C-cells based on a series of quail-chick chimaera lineage tracing experiments (Le Douarin and Le Lièvre, 1970; Polak et al., 1974). Following isotopic transplantation of quail neural tube into chick host embryos, they observed quail neural crest-derived cells within the host ultimobranchial gland that appeared to exhibit cytoplasmic secretory granules characteristic of C-cells (Le Douarin and Le Lièvre, 1970), and positive immunostaining with an anti-calcitonin antibody (Polak et al., 1974). These findings led to the assumption of a neural crest origin of C-cells in all vertebrates (including mammals), of C-cell-derived cancers like MTC (Gagel and Cote, 1998; Segura et al., 2018; Master and Burns, 2023) and, more broadly, of other biochemically similar neuroendocrine cell types [the amine precursor uptake and decarboxylation (APUD) series] that are distributed throughout the body in disparate organs and tissues (Pearse, 1969; Pearse and Polak, 1971a,b, 1974).

However, in the decades following reports of a neural crest origin of avian C-cells, corroborating evidence for a neural crest origin of mammalian C-cells remained scant. Kameda and colleagues tested for a neural crest contribution to C-cells in mouse using Connexin43-*lacZ* or Wnt1-Cre lineage tracing and found none – rather, they reported that developing mouse C-cells express E-cadherin (a common marker of epithelial cell types; Kameda et al., 2007). More recently, Johanson et al. demonstrated unequivocally using Sox17-Cre lineage tracing that mouse C-cells derive from pharyngeal endoderm, and not from the neural crest (Johansson et al., 2015). The distinct germ layer origins of C-cells in mouse and chick could indicate that these cell types are not homologous (i.e. that mammals and birds independently evolved calcitonin-secreting neuroendocrine cells in their thyroid glands and UBs, respectively) or that C-cells have undergone a radical lineage shift – from endoderm to neural crest, or vice versa – during tetrapod evolution.

Although it is now recognized that many other neuroendocrine cell types within Pearse's APUD series have non-neural crest embryonic origins (e.g. pancreatic endocrine cells, pulmonary neuroendocrine cells and gut enterochromaffin cells; Andrew, 1974; Andrew et al., 1983; Kuo and Krasnow, 2015), a conserved neural crest origin of C-cells remains cemented in the medical literature, despite a dearth of data on the embryonic origin of C-cells from other taxa. Here, we resolve the ancestral embryonic origin of C-cells using *in situ* gene expression analysis and cell lineage tracing in a range of vertebrate model systems. We revisit the embryonic origin of avian C-cells in the chick and find that, although neural crest cells do contribute connective tissue to the UBs, they do not give rise to calcitonin-expressing C-cells. Rather, chick C-cells derive from endoderm, as in the mouse. We also find that an endodermal origin of C-cells is conserved in a ray-finned bony fish (zebrafish) and in a cartilaginous fish (the little skate, *Leucoraja erinacea*). Finally, we test for calcitonin expression in two invertebrate chordate taxa that lack neural crest cells – the ascidian *Ciona intestinalis* and the amphioxus *Branchiostoma lanceolatum* – and we identify putative C-cell homologs within their endodermally-derived pharyngeal linings. These findings point to an ancestral and conserved endodermal origin of C-cells within chordates and broaden the repertoire of endodermal cell types in the ancestral chordate.

## RESULTS

### Chick C-cells derive from endoderm, and not neural crest

**Fig. 1.**
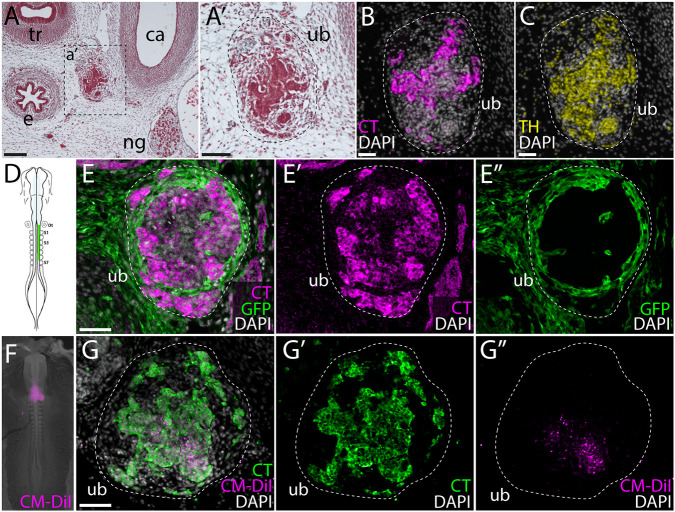
**Chick C-cells derive from endoderm, not neural crest**. (A,A′) At E10, the chick UB is located between the esophagus and the carotid artery, at the level of the nodose ganglion. (B,C) Chick C-cells co-express calcitonin (CT) (B) and tyrosine hydroxylase (TH) (C). (D) Neural crest lineage tracing was performed by isotopic unilateral grafting of GFP^+^ neural fold into a wild-type host embryo. In this example, a graft was performed with neural fold from the otic vesicle to somite 7. (E-E″) In grafted embryos, we recovered GFP^+^ cells in and around the UB, but we observed no colocalization of GFP and CT. (F) Endodermal lineage tracing was performed by microinjecting CM-DiI into the pharyngeal cavity of chick embryos at Hamburger Hamilton stage 11. (G-G″) In 3/5 labeled embryos, we recovered CM-DiI within the UB at E8.5, with colocalization of CM-DiI and CT indicating endodermal origin of chick C-cells. Dashed lines indicate the ultimobranchial body. ca, carotid artery; e, esophagus; ng, nodose ganglion; ot, otic vesicle; S1-S7, somites 1-7; tr, trachea; ub, ultimobranchial body. Scale bars: 100 μm (A); 50 μm (A′); 25 μm (B,C,E,G).

Considering that recent genetic lineage tracing data showed an endodermal origin of C-cells in mouse, we decided to revisit the germ layer origin of avian C-cells. In embryonic day (E)10 chick embryos, the UBs are located between the esophagus and the carotid artery, at the level of the nodose ganglion (Fig. 1A,A′), and chick C-cells express both calcitonin (Fig. 1B) and tyrosine hydroxylase (Fig. 1C; Fig. S1). The neural crest origin of chick C-cells was previously reported based on cell lineage tracing using chick-quail chimeras (Le Douarin and Le Lièvre, 1970; Pearse and Polak, 1971a,b; Polak et al., 1974). We carried out similar experiments, labeling pre-migratory cranial and vagal neural crest cells by unilaterally grafting cranial and vagal-level neural fold from GFP^+^ transgenic donor chick embryos (McGrew et al., 2008) into wild-type hosts. Grafted embryos were grown to E8.5-E10, and then sectioned and immunostained for GFP and calcitonin. We analyzed three embryos that received grafts of GFP^+^ neural fold from the otic vesicle to somite 1, and three embryos that received grafts of GFP^+^ neural fold from the otic vesicle to somite 7 (Fig. 1D). In all six embryos, we recovered GFP^+^ cells inside and around the UB but no colocalization of GFP and calcitonin in any embryos (Fig. 1E-E″; Fig. S2; Table 1). This strongly suggests that chick C-cells do not derive from the neural crest. In Fig. 1E, apparent calcitonin expression is also present in a nerve adjacent to the UB. This staining could reflect calcitonin-expressing C-cells within the nerve (see Discussion), or cross-reaction of our anti-calcitonin antibody with calcitonin gene-related peptide in the nerve.

**Table 1.**
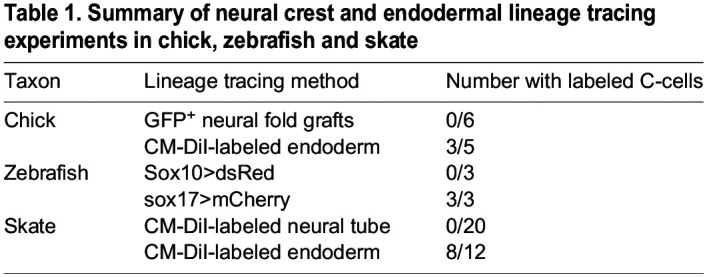
Summary of neural crest and endodermal lineage tracing experiments in chick, zebrafish and skate

To test for an alternative endodermal origin of C-cells in the chick, we microinjected the lipophilic dye CM-DiI into the pharyngeal cavity of chick embryos at Hamburger Hamilton stage 11 (Fig. 1F) to label the endodermal epithelium that lines the pharyngeal cavity. We then grew injected embryos to E8.5 and tested for colocalization of CM-DiI and calcitonin by immunofluorescence. In 3/5 labeled embryos, we recovered CM-DiI-labeled C-cells within the UBs (Fig. 1G-G″; Table 1), indicating an origin of these cells from pharyngeal endoderm. So, although the UB is surrounded by and contains some neural crest-derived cells, we find no evidence that C-cells themselves derive from the neural crest in chick. Instead, as in mouse, we find that chick C-cells have an endodermal origin.

### Zebrafish C-cells derive from endoderm

**Fig. 2.**
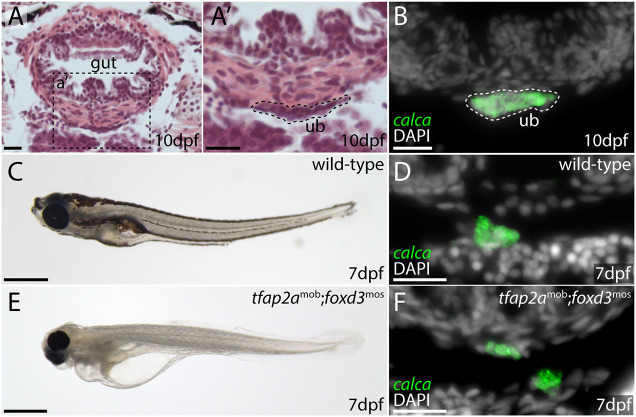
**C-cells of the larval zebrafish develop in the absence of neural crest.** (A-B) The UB of a 10 dpf zebrafish is located between the gut and the heart, at the axial level of the sinus venosus (A). It is recognizable as a distinct cluster of cells beneath the muscle layer of the gut (A′), and by its expression of *calca* (B). Dashed lines indicate the ultimobranchial body. (C,D) 7 dpf wild-type zebrafish (C) possess *calca*^+^ C-cells within their UB (D). (E,F) 7 dpf *tfap2a*^mob^;*foxd3*^mos^ mutants (E) lack neural crest cells, but still possess *calca*^+^ C-cells in their UB (F). ub, ultimobranchial body. Scale bars: 20 μm (A,A′,B,D,F); 1 mm (C,E).

We next sought to resolve the embryonic origin of C-cells in a ray-finned fish outgroup to the tetrapods. This would allow us to infer the ancestral germ layer origin of C-cells for bony vertebrates. In the larval zebrafish, the UBs meet at the ventral midline beneath the ventral wall of the esophagus, at the axial level of the sinus venosus (Fig. 2A,A′). C-cells within the UB are recognizable by their expression of *calca* (with splice variants of *calca* encoding calcitonin and calcitonin gene-related peptide) by mRNA *in situ* hybridization (ISH) (Fig. 2B). We analyzed *tfap2a^mob^;foxd3^mos^* zebrafish, which lack neural crest derivatives (Wang et al., 2011), to determine whether these mutants also lack C-cells. We first tested for *calca* expression in cells of the developing UB at 7 days post-fertilization (dpf) in wild-type siblings, and we observed *calca*^+^ cells within the UB of all larvae (Fig. 2C,D; *calca*^+^ cells found in *n*=6/6 individuals analyzed). At 7 dpf, *tfap2a^mob^;foxd3^mos^* mutants exhibit a distinct lack of pigment and craniofacial malformations (Fig. 2E), owing to an absence of neural crest-derived melanocytes and skeletal tissues, respectively, but we found that these mutants nevertheless possessed *calca*^+^ cells within their UB (Fig. 2F; *calca*^+^ cells found in *n*=6/6 individuals analyzed). The presence of *calca*^+^ cells in the UBs of mutants lacking neural crest cells indicates a likely non-neural crest origin of C-cells in zebrafish.

**Fig. 3.**
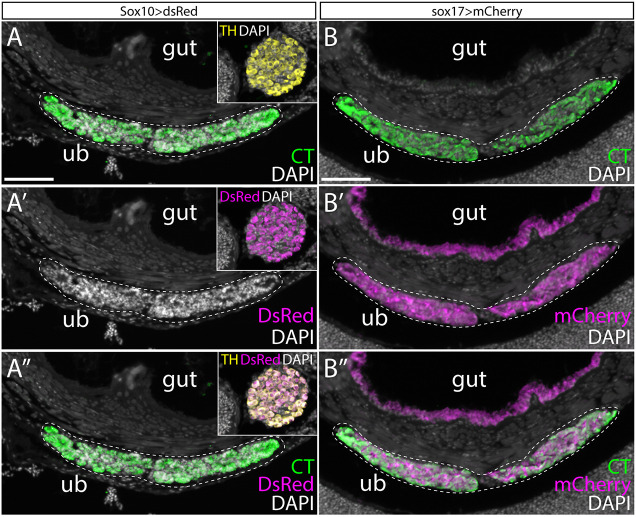
**Zebrafish C-cells derive from pharyngeal endoderm, not neural crest.** (A-A″) The UB of adult zebrafish stains positively for calcitonin (CT) by immunofluorescence (A). The Sox10>dsRed line labels all neural crest derivatives with DsRed. We found no DsRed^+^ cells within the UB of adult fish of this line, despite strong labeling of other neural crest-derived strictures on the same sections – e.g. Tyrosine Hydroxylase (TH)-positive neurons of the sympathetic ganglia (see inset boxes in A-A″). (B-B″) sox17>mCherry zebrafish embryos were treated with 4OHT during gastrulation to indelibly label the endodermal lineage with mCherry, and then grown to adult. We observed complete colocalization of mCherry and CT within the UB of adult fish, indicating endodermal origin of zebrafish UB C-cells. Dashed lines indicate the ultimobranchial body. ub, ultimobranchial body. Scale bars: 50 μm (A,B).

Next, we employed genetic cell lineage tracing to test for a neural crest contribution to C-cells in the ultimobranchial gland of zebrafish. Although we were able to detect transcription of *calca* in the UB of larval zebrafish at 7-10 dpf, we were unable to detect expression of calcitonin protein at these stages. We therefore opted to test for neural crest contributions to UB C-cells in adult zebrafish when Calcitonin expression is readily detectable by immunofluorescence. We used Sox10:Cre; actab2:loxP-BFP-STOP-loxP-dsRed (Sox10>dsRed) fish, which enabled the permanent labeling of neural crest cell derivatives shortly after their differentiation at 10 h post-fertilization (hpf) (Kague et al., 2012; Fabian et al., 2022). Fish were raised to adulthood (150-210 dpf) and sections through the entire UB were co-stained for the dsRed reporter of neural crest lineage and calcitonin by immunofluorescence. In 3/3 fish examined, we observed no dsRed^+^ cells within the UBs (Fig. 3A,A′; Table 1), despite strong labeling of other neural crest derivatives within the same sections (e.g. TH^+^ neurons of the sympathetic ganglia – see inset boxes in Fig. 3A-A″). These observations further support a non-neural crest origin of C-cells in zebrafish.

Having excluded neural crest cells as progenitors for C-cells, we tested for an endodermal origin of zebrafish C-cells. To specifically mark endodermal lineages in zebrafish, we employed sox17:CreERT2;ubi:loxP-eGFP-STOP-loxP-mCherry (sox17>mCherry) fish and treated them with 4-hydroxytamoxifen (4OHT) during gastrulation (4-6 hpf) to induce loxP recombination and indelible labeling of the endodermal lineage with mCherry (Fabian et al., 2020). Fish were raised to adulthood (150-210 dpf) and sections through the UBs were co-stained for the mCherry reporter of endodermal lineage and Calcitonin by immunofluorescence. We found consistent co-expression of the mCherry reporter of endodermal lineage and calcitonin throughout the UBs in 3/3 fish analyzed (Fig. 3B-B″; Table 1), indicating an endodermal origin of C-cells in zebrafish. When considered alongside data from mouse and chick, this finding indicates that C-cells ancestrally derive from endoderm in bony vertebrates.

### Skate C-cells derive from endoderm

**Fig. 4.**
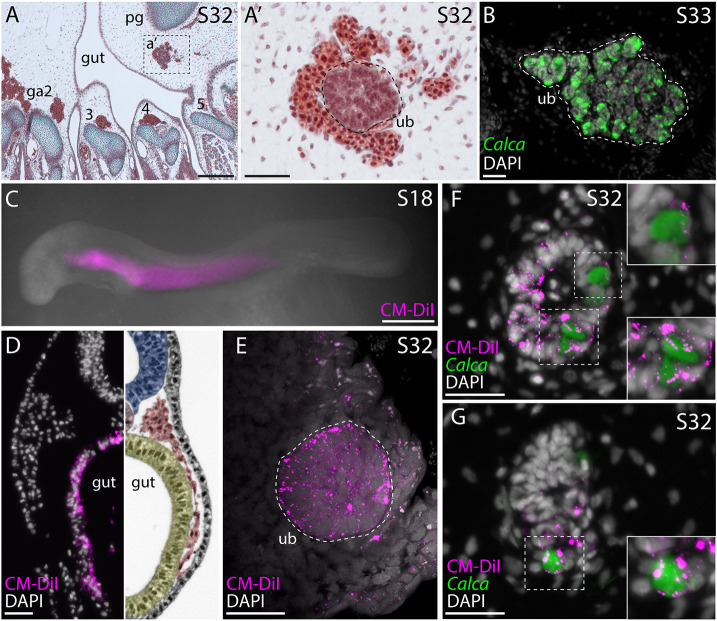
**Skate C-cells derive from pharyngeal endoderm.** (A,A′) In a stage (S)32 skate embryo, the developing UB is located within connective tissue between the fifth gill arch and the pectoral girdle. (B) The UB of a stage 33 skate embryo, with *Calca* expression in C-cells. (C,D) Microinjection of CM-DiI into the pharyngeal cavity of a S18 skate embryo (C) specifically labels the pharyngeal endoderm (D). (E) Maximum intensity projection of the UB of a S32 skate embryo that received CM-DiI labeling of pharyngeal endoderm at S18. There is abundant CM-DiI labeling throughout the UB. Dashed lines indicate the ultimobranchial body. (F,G) Colocalization of CM-DiI and *Calca* expression within the UB of S32 skate embryos indicate endodermal origin of C-cells in the skate. Insets show magnification of boxed areas. ga2-5, gill arches 2-5; pg, pectoral girdle; ub, ultimobranchial body. Images in panels C and D are reproduced from Rees et al. (2023) under the terms of a CC-BY 4.0 license. Scale bars: 200 μm (A); 50 μm (A′); 25 μm (B,D-G); 500 μm (C).

All extant jawed vertebrates belong to one of two lineages: bony vertebrates (including bony fishes and tetrapods) and cartilaginous fishes (sharks, skates, rays and holocephalans). To test whether an endodermal origin of C-cells is conserved in cartilaginous fishes, we carried out a series of cell lineage tracing experiments in a cartilaginous fish outgroup to the bony vertebrates, the little skate (*L. erinacea*) (Gillis et al., 2022). In skate embryos, UBs are located between the caudal gill arches and the pectoral girdle (Fig. 4A,A′), and as in bony vertebrates, skate C-cells within the UBs are marked by expression of calcitonin (Fig. 4B). To test whether skate C-cells derive from endoderm, we microinjected the pharyngeal cavity of neurula-stage skate embryos with the lipophilic dye CM-DiI (Fig. 4C). By microinjecting CM-DiI into the pharyngeal cavity before pharyngeal pouches and gill slits have formed, we can broadly label pharyngeal endoderm without contaminating adjacent tissues (Fig. 4D) (Gillis and Tidswell, 2017; Rees et al., 2023). Most importantly, at the stage in which we conduct our pharyngeal endodermal labeling experiments, pre-migratory neural crest cells are specified in the dorsal neural tube, but have not yet begun to migrate (Gillis et al., 2017), thus precluding the possibility of inadvertent neural crest cell contamination with our CM-DiI labeling. Injected embryos were left to develop until stage 32 (∼8-10 weeks post-injection), by which point the UBs are differentiating.

Embryos that received pharyngeal endodermal labeling with CM-DiI showed abundant CM-DiI-retention throughout the developing UBs (Fig. 4E). To specifically test for an endodermal origin of C-cells, we sectioned 12 stage 32 embryos that had received CM-DiI-labeling of pharyngeal endoderm and found that eight embryos showed colocalization of CM-DiI with *Calca* expression within the UB (Fig. 4F,G; Table 1). We also examined 15 skate embryos that received microinjection of CM-DiI into the neural tube at neurula stage (to label pre-migratory neural crest cells) and we found no CM-DiI-labeled cells in the UB of any of these embryos (Table 1) (Sleight and Gillis, 2020). These findings indicate that C-cells derive from pharyngeal endoderm in the skate. When considered alongside data from mouse and zebrafish, this finding supports an ancestral endodermal origin of C-cells for jawed vertebrates.

### Identification of putative endoderm-derived C-cells in non-vertebrate chordates

**Fig. 5.**
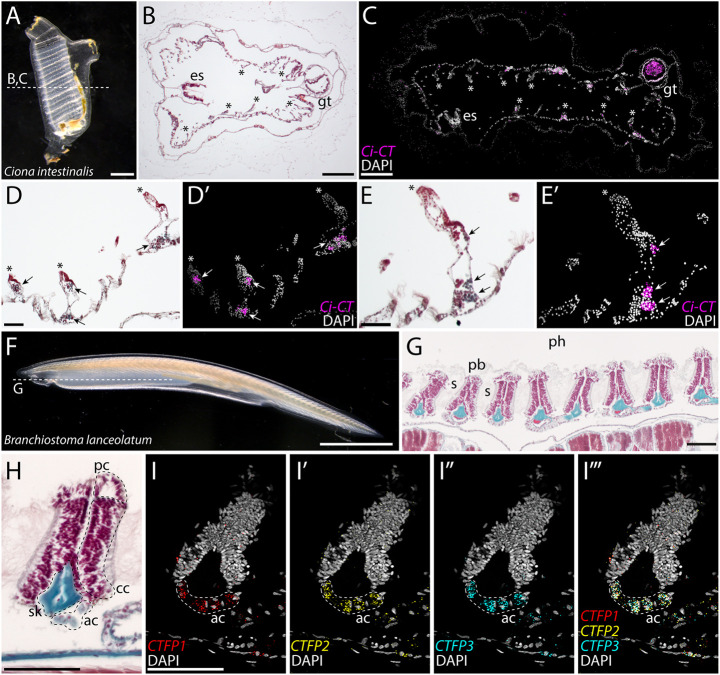
**Putative C-cell homologs within the pharynx of invertebrate chordates.** (A,B) Adult *Ciona intestinalis* (A). Sections in the plane indicated by the dashed line reveal a branchial sac that is perforated by slits, with an endostyle on one side and the gut on the other (B). (C) HCR for *Ci-CT* reveals expression in the endodermally-derived wall of the branchial sac (note autofluorescence within the gut). Asterisks in B and C indicate the papillae of the branchial sac. (D-E′) Expression of *Ci-CT* localizes to distinct clusters of cells within the papilla of the branchial sac. No expression was observed within the endostyle. (F,G) An adult amphioxus, *Branchiostoma lanceolatum* (F). Sections in the plane indicated by the dashed line reveal a pharyngeal cavity containing a series of pharyngeal bars separated by pharyngeal slits (G). A lower magnification image of the section in G is shown in Fig. S5A. (H) Each pharyngeal bar consists of atrial cells, ciliated cells and pharyngeal cells, and is supported by an acellular skeletal rod. (I) HCR for amphioxus *CTFP1*-*3* reveals co-expression of all three paralogs within the atrial cells of each pharyngeal bar. Dashed lines indicate CTFP-expressing atrial cells. ac, atrial cells; cc, ciliated cells; es, endostyle; gt, gut; pb, pharyngeal bar; pc, pharyngeal cells; s, pharyngeal slit; sk, skeletal rod. Scale bars: 1 mm (A); 250 μm (B,C); 50 μm (D,E); 2 mm (F); 75 μm (G-I).

Tunicates and cephalochordates are invertebrate chordates that lack a bona fide neural crest, but that share many other body plan features with vertebrates, including an endodermally-derived pharynx (Lowe et al., 2015). Previous studies reported granulated and argyrophilic cell types in the endostyle of the tunicate *Styela clava* (Thorndyke and Probert, 1979) and in the stomach of the tunicate *C. intestinalis* (Fritsch et al., 1980) that showed reactivity when stained with an anti-human calcitonin antibody. *C. intestinalis* has a single calcitonin-like gene (*Ci-CT*), and expression of this gene has been previously reported in the *C. intestinalis* pharynx by wholemount mRNA ISH (Sekiguchi et al., 2009; Hamada et al., 2011; Satake and Sasakura, 2023). To expand on previous reports, we tested for the expression of *Ci-CT* in paraffin sections of the entire adult *C. intestinalis* using mRNA ISH by chain reaction (HCR). The adult life stage of *C. intestinalis* (Fig. 5A) possesses an endodermally-derived branchial sac (Hirano and Nishida, 2000) that is perforated by numerous pharyngeal slits. This branchial sac is bordered on one side by an endostyle and on the other side by the gut (Fig. 5B) and bears inward-directed epithelial elaborations called papillae (Osugi et al., 2020). Consistent with previous studies (Fritsch et al., 1980), we observed Ci-CT-expression in clusters of cells around the stomach of *C. intestinalis* (Fig. S3), and we additionally observed widespread expression in the lining of the pharynx (Fig. 5C). This pharyngeal expression localized to histologically distinct cells within the papillae of the branchial sac (Fig. 5D-E′). These *Ci-CT*-expressing cells also report positively for the expression of *prohormone convertase 2*, an endopeptidase that converts prohormones and neuropeptide precursors to their active forms (Osugi et al., 2020), pointing to a likely neuroendocrine function for these cells. Contrary to previous studies (Thorndyke and Probert, 1979; Sekiguchi et al., 2009), we found no expression of *Ci-CT* in the endostyle of adult *C. intestinalis*.

The pharynx of adult amphioxus (Fig. 5F) consists of a series of ∼50 pharyngeal slits that are separated by bars and is bordered ventrally by an endostyle (Fig. 5G). Pharyngeal bars have a thick surface epithelium divided into three portions: atrial cells that line the lateral surface of the bars, ciliated cells that are located on the medial side of each bar, and pharyngeal cells that line the bar between the atrial and ciliated cells (Fig. 5H). Pharyngeal bars are also supported internally by a skeletal rod, consisting of an expanded acellular collagenous matrix (Baskin and Detmers, 1976). Cephalochordates have three calcitonin genes encoding calcitonin family proteins (CTFPs), and these three genes are expressed in different combinations in neurons of the amphioxus larva and adult, and in isolated cells within the adult midgut and hindgut (Sekiguchi et al., 2016; Sekiguchi, 2018; Gattoni et al., 2023 preprint). We identified the complete sequence and genomic location of the three CTFP genes in *B. lanceolatum*. Phylogenetic analysis and the close arrangement of CTFP genes on the same chromosome together indicate that CTFPs duplicated independently in the cephalochordate lineage (Fig. S4). We generated probes for all three CTFP paralogs and tested for their expression in longitudinal sections of the adult *B. lanceolatum* pharynx using *in situ* HCR. We found co-expression of all three CTFP paralogs in the atrial cells of each pharyngeal bar (Fig. 5I-I‴; Fig. S5), and we detected no expression of any CTFP paralog in the endostyle. These findings point to a calcitonin-expressing cell type within the endodermally-derived pharyngeal lining of tunicates and cephalochordates, and to an ancient, pre-vertebrate endodermal origin of calcitonin-secreting neuroendocrine cells.

## DISCUSSION

### Conservation of C-cell embryonic origin within jawed vertebrates

Avian C-cells exhibit some striking and unique neural-like features that are not shared with those of other vertebrate taxa. For example, chick C-cells may be directly innervated by nerve fibers originating from the vagus and recurrent laryngeal nerves (Kameda et al., 1988), unlike mammalian C-cells, which receive no direct innervation. Chick C-cells are also distinct from the C-cells of other vertebrates in their co-expression of the neural markers tyrosine hydroxylase and enkephalin, and in their possession of elongated cell processes that contact those (or somata) of adjacent C-cells during earlier stages of their differentiation (Kameda, 1991; Kameda et al., 1993). The discovery of calcitonin-positive C-cells within the nerve bundles that invade the UB and the adjacent nodose ganglion (Kameda, 1989), along with evidence of a neural crest origin from previous lineage tracing experiments (Le Douarin and Le Lièvre, 1970; Pearse and Polak, 1971a,b; Polak et al., 1974), led to a model in which neural crest progenitor cells traveling from the nodose ganglion and the vagus nerve surround and invade the UB before differentiating into C-cells by E10 (Kameda, 1995). Importantly, the epithelial cells that line the cystic cavities of the post-hatching and adult chicken UB also stain positively for calcitonin (Kameda, 1984). Based on the assumption that the latter derive from the endodermal epithelium of the UB, it has been suggested that avian C-cells may have a dual embryonic origin from neural crest and endoderm (Kameda, 1995; 2017), with neural crest-derived C-cells possibly representing an avian innovation.

Although histological snapshots can provide invaluable data on the progression of differentiation and marker expression within tissues through development, they cannot determine cell lineage. Our study finds that chick C-cells do not derive from the neural crest, and that previous lineage tracing experiments using quail-chick chimeras likely recovered a neural crest contribution to connective tissue or other cell types within the parenchyma of the UB, rather than to the calcitonin-expressing C-cells. The challenges of sequential imaging of cell type markers and cell lineage (quail versus chick), using formaldehyde-induced fluorescence/immunohistochemistry and Feulgen staining, respectively, could account for earlier interpretations of UB C-cells as a derivative of the neural crest. We performed cell lineage tracing experiments by grafting neural folds from GFP^+^ chick embryos into wild-type hosts and, unlike earlier experiments, this approach allowed us to image cell lineage and cell type marker expression simultaneously. We found no evidence of a neural crest contribution to calcitonin-expressing C-cells, but rather found, by direct labeling of pharyngeal endoderm with the lipophilic dye CM-DiI, that this tissue gives rise to the calcitonin-expressing C-cells of the UB at E10. Immuno-electron-microscopical studies of the chick UB reported three types of neuroendocrine cells (types-I, II and III), based on differences in the size, nature and contents of their secretory granules (Kameda et al., 1993). Immunolabeling using the protein A-colloidal gold method revealed that calcitonin colocalized with the secretory granules of type-I and -II cells, whereas enkephalin colocalized with the granules of type-II and -III cells. It was noted that the type-III neuroendocrine cells (which express enkephalin, but not calcitonin) resemble C-cells in an earlier state of differentiation (Kameda, 1991; Kameda et al., 1993). Rather than representing a C-cell precursor, we suggest instead that these cells could represent an additional, avian-specific neuroendocrine cell type, distinct from the endoderm-derived calcitonin-expressing C-cells, and that this cell type could derive from neural ectoderm or the neural crest.

The endodermally-derived C-cells in mammals have a well-documented propensity for migration, despite their embryonic origin within an epithelium. As discussed above, mammalian C-cells develop from cells of the transient UB, eventually invading and dispersing within the developing thyroid primordium. Additionally, in cat, rabbit, goat and dog, C-cells may be found not only in the thyroid gland, but also in varying numbers within the neighboring parathyroid and thymus glands (Kameda, 1971; 1981). It is therefore plausible that calcitonin-expressing C-cells within the avian vagus nerve and nodose ganglion could reflect the migration of C-cells into, rather than an origin from, those neural tissues. Finally, it has become increasingly evident that endoderm has the capacity to differentiate into cell types long thought to derive exclusively from other embryonic germs layers. Endoderm can give rise to tooth ameloblasts (Soukup et al., 2008), neuroendocrine cells of the anterior pituitary (Fabian et al., 2020) and even neurons (Wei et al., 2011; Nakanishi et al., 2012) – all cell types that are widely regarded as ectodermal derivatives. These observations stress the need for cell lineage tracing, in addition to data on cell phenotypes (e.g. morphology and gene expression), to confidently infer the germ layer origin of a cell type.

Before this study, no data were available on the embryonic origin of vertebrate C-cells outside of tetrapods, though an endodermal origin has been assumed (Kameda, 2017). In zebrafish, the UB consists of several small epithelial follicles, and sits beneath the circular musculature of the esophagus immediately rostral to the sinus venosus (Yamane, 1978), whereas in batoid elasmobranch fishes (skates and rays), the UB forms initially as a single large follicle but subsequently takes the form of a solid mass of cells (Kameda, 2017, and this study). Both teleost and batoid UBs express calcitonin mRNA and/or protein (McMillan et al., 1976; Takei et al., 1991; Hidaka and Suzuki, 2004; Alt et al., 2006). Here, we formally demonstrate, by lineage tracing, that the UB C-cells of zebrafish and skate both derive from endoderm, with no evidence of a neural crest contribution. Alongside lineage tracing data from mouse (Kameda et al., 2007; Johansson et al., 2015) and chick (this study), these findings point to a broadly conserved endodermal origin of C-cells across jawed vertebrates (tetrapods, bony fishes and cartilaginous fishes).

### A pre-vertebrate origin of endodermally-derived C-cells

**Fig. 6.**
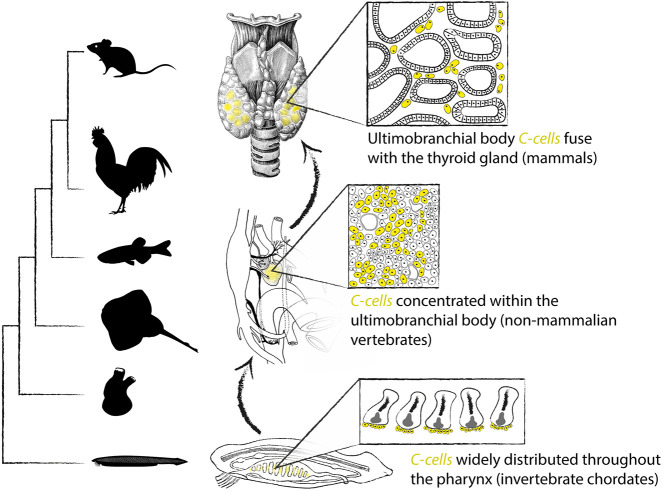
**Evolution and homology of chordate C-cells.** C-cells were broadly distributed throughout the endoderm-derived lining of the pharynx in the last common ancestor of chordates. These C-cells then became localized into a discrete ultimobranchial body in non-mammalian vertebrates, with that ultimobranchial body fusing with the thyroid gland of mammals.

We additionally report discrete calcitonin-positive cell types (putative C-cell homologs) that reside within the endodermally-derived pharyngeal lining of the invertebrate chordates *C. intestinalis* and *B. lanceolatum*. These observations support a pre-vertebrate origin of an endodermally-derived calcitonin-expressing neuroendocrine cell, broaden the ancestral chordate endodermal cell type repertoire to include a new neuroendocrine lineage, and establish homology of C-cells throughout chordate phylogeny. There is deep conservation across deuterostomes of a core pharyngeal endodermal transcriptional program (Simakov et al., 2015). Genes encoding transcription factors such as Pax1/9 (Peters et al., 1998), Six1 (Zou et al., 2006) and Eya1 (Xu et al., 2002) that specify glandular tissues (e.g. the thymus, parathyroid and UBs) are expressed within the caudal pharyngeal endodermal pouches of vertebrate embryos but are expressed iteratively in all developing pharyngeal pores of amphioxus (Kozmik et al., 2007; Liu et al., 2015) and hemichordates (Gillis et al., 2012a). The iterative deployment of this conserved transcriptional program throughout pharyngeal development could account for the broader distribution of some neuroendocrine cell types, such as C-cells, within pharyngeal endodermal derivatives of invertebrate chordates, with these cells subsequently becoming localized to specific glandular structures with tissue elaboration and enhanced regionalization of the vertebrate pharynx (Fig. 6).

## MATERIALS AND METHODS

### Zebrafish lines

The following zebrafish (*Danio rerio*) lines were used: Tg(Mmu.Sox10-Mmu.Fos:Cre)^zf384Tg^ (Kague et al., 2012), Tg(actab2:loxP-BFP-STOP-loxP-dsRed)^sd2Tg^ (Kobayashi et al., 2014), Tg(-5.0sox17:creERT2; myl7:DsRed)^sid1Tg^ (Hockman et al., 2017), Tg(-3.5ubb:loxP-eGFPloxP-mCherry)^cz1701Tg^ (Mosimann et al., 2011) and *tfap2a^mob^;foxd3^mos^*(Wang et al., 2011). To induce Cre recombination in the endoderm, Tg(-5.0sox17:creERT2;myl7:DsRed)^sid1Tg^; Tg(-3.5ubb:loxP-GFPloxP-mCherry)^cz1701Tg^ embryos were incubated in embryo media containing 5 μM (Z)−4-hydroxytamoxifen (Sigma-Aldrich H7904) starting at 4-4.5 hpf and then washed several times in fresh embryo media at 6 hpf. For lineage tracing experiments, only preselected animals with high conversion were used. Experiments using all zebrafish lines were conducted according to protocols approved by the Institutional Animal Care and Use Committees in facilities accredited by the Association for Assessment and Accreditation of Laboratory Animal Care International (AAALAC). All zebrafish were fixed overnight at 4°C in 4% paraformaldehyde in phosphate-buffered saline (PBS), rinsed in PBS, and dehydrated into methanol before analysis.

### Skate embryos

Skate (*L. erinacea*) embryos were obtained from the Marine Resources Center at the Marine Biological Laboratory (MBL; Woods Hole, MA, USA), were reared and staged as described in Gillis et al. (2022). All skate experiments were conducted according to protocols approved by the Institutional Animal Care and Use Committee of the MBL. Endodermal lineage tracing was performed by microinjection of CellTracker CM-DiI (1,1′-dioctadecyl-3,3,3′3′-tetramethylindocarbocyanine perchlorate) into the pharyngeal cavity of stage 18 skate embryos with a pulled glass needle. CM-DiI was prepared as previously described (Gillis et al., 2012a,b). Skate embryos were euthanized with an overdose of MS-222 (1 g/l in seawater; Sigma-Aldrich) and all embryos were fixed overnight at 4°C in 4% paraformaldehyde in PBS, rinsed in PBS and dehydrated into methanol before analysis.

### Chicken embryos

Experiments using chicken (*Gallus gallus domesticus*) embryos were conducted in accordance with the UK Animals (Scientific Procedures) Act 1986. Fertilized wild-type chicken eggs were obtained from Henry Stewart and Co., Norfolk, UK. Fertilized GFP-transgenic chicken eggs (McGrew et al., 2008) were obtained from the Roslin Institute Transgenic Chicken Facility (Edinburgh, UK), which is funded by Wellcome and the Biotechnology and Biological Sciences Research Council. Wild-type and GFP-transgenic eggs were incubated in a humidified atmosphere at 38°C for ∼1.5 days to reach 6-11 somites and embryos visualized as previously described (Dude et al., 2009), using filtered PBS instead of Ringer's solution. To label pre-migratory vagal neural crest cells, neural fold between the level of the otic vesicle and the caudal end of somite 1 (s1) (unilaterally) or somite 6 (s6) (unilaterally) was grafted isotopically from GFP-transgenic donors to wild-type hosts using a pulled glass needle. Chick embryos were fixed overnight at 4°C in 4% paraformaldehyde in PBS, rinsed in PBS and dehydrated into methanol before analysis.

### *Branchiostoma* and *Ciona* collection

Adults of the European amphioxus (*B. lanceolatum*) were collected in Banyuls-sur-Mer, France and maintained in a custom-made facility at the Department of Zoology, University of Cambridge (Benito-Gutierrez et al., 2013). Animals were anesthetized with a 30 min immersion in 0.015% tricaine methanesulphonate (MS-222), divided into rostral and caudal halves using a razor blade, and fixed in 4% paraformaldehyde for 24 h at 4°C, as previously described (Andrews et al., 2020). Adults of the sea squirt *C. intestinalis* were kept in tanks within the amphioxus facility at the Department of Zoology, University of Cambridge. Small adult ascidians were detached from the tanks, relaxed with menthol crystals dissolved in seawater and fixed in 4% paraformaldehyde for 24 h at 4°C. The anterior half of fixed amphioxus and whole *Ciona* young adults were then processed for sectioning and ISH.

### Sequence analysis

The complete prepropeptide sequences of multiple vertebrate calcitonin and calcitonin gene-related peptides, as well as of *Ciona*, amphioxus and starfish calcitonin-type prepropeptides (Table S1) were aligned using MAFFT (Katoh and Standley, 2013) and trimmed using trimAl (Capella-Gutiérrez et al., 2009). Neighbor joining molecular phylogenetics was carried out with seaview (Gouy et al., 2021), using the related neuropeptide adrenomedullin as outgroup, and the resulting tree visualized with FigTree (http://tree.bio.ed.ac.uk/software/figtree/).

### Sectioning and staining

Before embedding, adult zebrafish were decalcified in Morse solution (10% w/v sodium citrate dihydrate and 25% v/v formic acid in DEPC water) for 24 h at room temp with gentle agitation. All tissue samples were cleared with Histosol (National Diagnostics) for 3×20 min at room temperature, transitioned into 1:1 Histosol:Paraffin for 2×30 min at 60°C, then infiltrated with molten paraffin overnight at 60°C. After an additional 4×1 h paraffin changes, samples were embedded in peel-a-way molds (Sigma-Aldrich), left to set for 24 h and then sectioned at 7 μm on a Leica RM2125 rotary microtome. Sections were mounted on SuperFrost Plus charged glass slides. Histochemical staining with modified Masson's Trichrome was performed as described by Witten and Hall (2003).

### mRNA ISH on paraffin sections

Chromogenic mRNA ISH was performed on paraffin sections as previously described (O'Neill et al., 2007) with modifications according to Gillis et al. (2012a,b). Chromogenic ISH probes against zebrafish *calca* (GenBank DQ406589.1) and skate *Calca* (GenBank XM_055649884.1) were generated by *in vitro* transcription using standard methods. Third-generation HCR was performed as per Choi et al. (2018) following the protocol for formaldehyde-fixed, paraffin-embedded sections, with modifications according to Criswell and Gillis (2020). For chick and skate, probes, buffers, and hairpins were purchased from Molecular Instruments. HCR probe set lot numbers from Molecular Instruments are as follows: Chicken *Calca* (PRD684), Chicken *TH* (PRD683), Skate *Calca* (PRK855) and *Ciona* Ci-CT (PRI432). For *Branchiostoma*, HCR probes against *CTFP1* (GenBank PP830919), *CTFP2* (GenBank PP830920) and *CTFP3* (GenBank PP830921) were designed using the Özpolat Lab HCR probe generator (https://github.com/rwnull/insitu_probe_generator).

### Immunofluorescence on paraffin sections

All slides for immunofluorescence were dewaxed for 2×5 min in Histosol, rehydrated through a descending ethanol series, and washed 3×5 min in PBS+0.1% Triton X-100 (PBST). Antigen retrieval was performed by pre-warming slides in distilled water at 60°C for 5 min, then incubating in 10 mM tri-sodium citrate (pH 6.0) at 95°C for 25 min. Slides were then cooled at −20°C for 30 min and rinsed 3×5 min in PBST before blocking in 10% heat-inactivated sheep serum at room temperature for 60 min. Primary antibodies were applied underneath a parafilm coverslip, and slides were incubated in a humidified chamber overnight at 4°C. Following primary antibody incubation, slides were rinsed 3×10 min with PBST and then secondary antibodies were applied underneath a parafilm coverslip. Slides were incubated at room temperature for 4 h in the dark. Slides were then rinsed 3×10 min in PBST, then 3×30 min in PBST before coverslipping with Fluoromount G containing DAPI. Primary antibodies used were: mouse anti-mCherry (Abcam, ab125096; 1:250), mouse anti-GFP (Merck, SAB5300167; 1:250), rabbit anti-tyrosine hydroxylase (Merck, AB152; 1:250), rabbit-anti calcitonin (BMA Biomedicals, T-4026; 1:250), goat anti-mouse IgG (H+L) cross-adsorbed secondary antibody, Alexa Fluor 488 (Thermo Fisher Scientific, A11001; 1:500), goat anti-rabbit IgG (H+L) cross-adsorbed secondary antibody, Alexa Fluor 488 (Thermo Fisher Scientific, A11008; 1:500), goat anti-mouse IgG (H+L) cross-adsorbed secondary antibody, Alexa Fluor 633 (Thermo Fisher Scientific, A21050; 1:500) and goat anti-rabbit IgG (H+L) highly cross-adsorbed secondary antibody, Alexa Fluor 633 (Thermo Fisher Scientific, A21071; 1:500).

### Imaging and image processing

Images were taken on a Zeiss Axioscope A1 compound microscope with a Zeiss Colibri 7 fluorescence LED light source using a Zeiss Axiocam 305 color or 503 mono camera and ZenPro software. All figures were assembled using Adobe Creative Cloud. Images of chromogenic ISH were inverted and overlaid with corresponding DAPI images.

## Supplementary Material



10.1242/develop.202821_sup1 Supplementary information
