# Supplementary material for: A pre-vertebrate endodermal origin of calcitonin-producing neuroendocrine cells
Source: Development. 2024 Aug 7;151(20):dev202821. doi: 10.1242/dev.202821 (PMC11698069; doi:10.1242/dev.202821)
Supplement: Supplementary information [file develop-151-202821-s1.pdf]

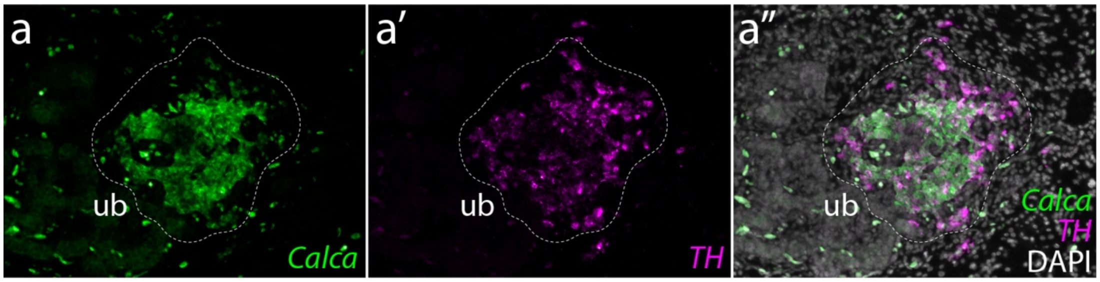

Fig. S1. Chick C-cells co-express *Calca* and *TH*. a) *Calca* and **a')** *TH* are **a")** largely co-expressed in the C-cells of the chick UB. Expression detected by multiplexed fluorescent mRNA *in situ* hybridization by chain reaction. *ub*, ultimobranchial body.

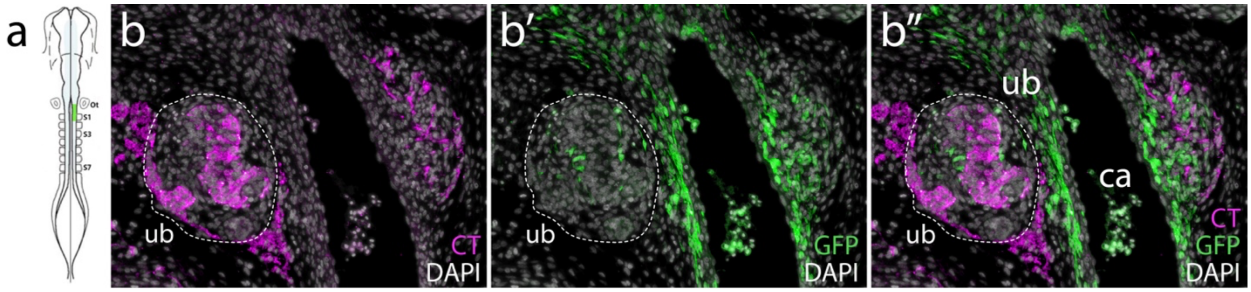

Fig. S2. Chick C-cells do not derive from the neural crest. Neural crest lineage tracing was performed by isotopic unilateral grafting of GFP+ neural fold into a wild-type host embryo. **a)** In this example, a graft was performed with neural fold from the otic vesicle to S1. **b)** Calcitonin (CT) is expressed in the C-cells of the UBU. **b')** GFP+ neural crest derived cells are recovered within and around the UB, but **b")** we observed no co-localisation of GFP and CT. *ub*, ultimobranchial body.

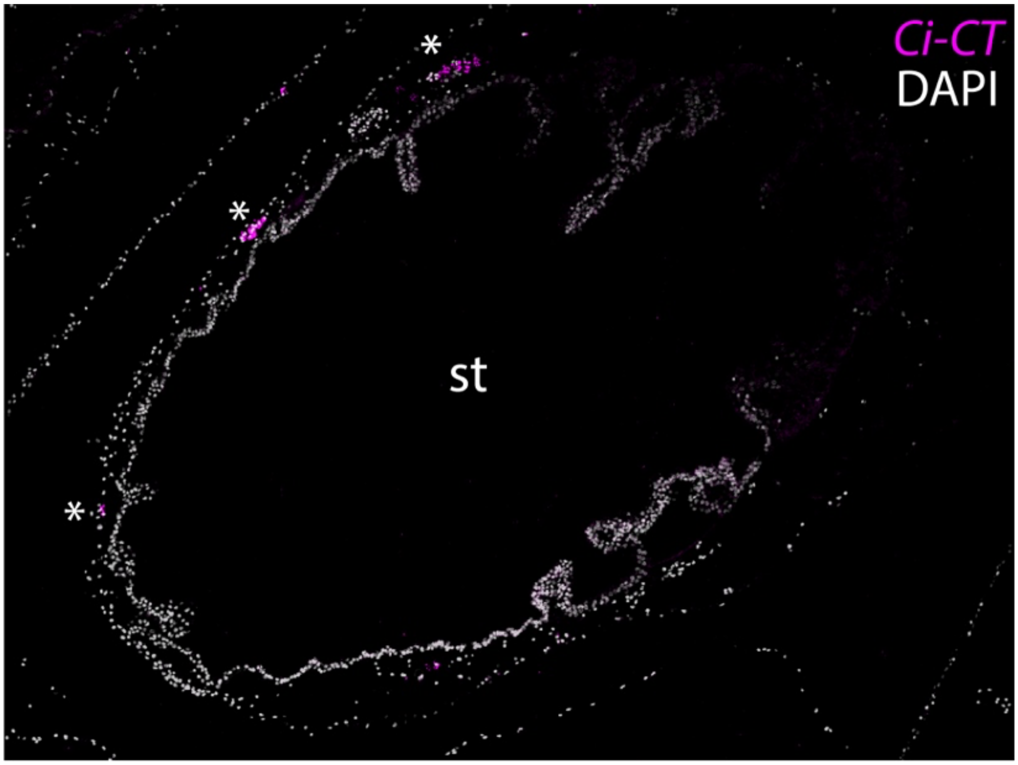

Fig. S3. Ci-CT-expressing cells around the stomach of *Ciona intestinalis*. Consistent with a previous report[50], we observed cells (*) that express Ci-CT around the stomach of adult *C. intestinalis*. Expression detected by mRNA *in situ* hybridization by chain reaction on a transverse section through adult *C. intestinalis*. *st*, stomach.

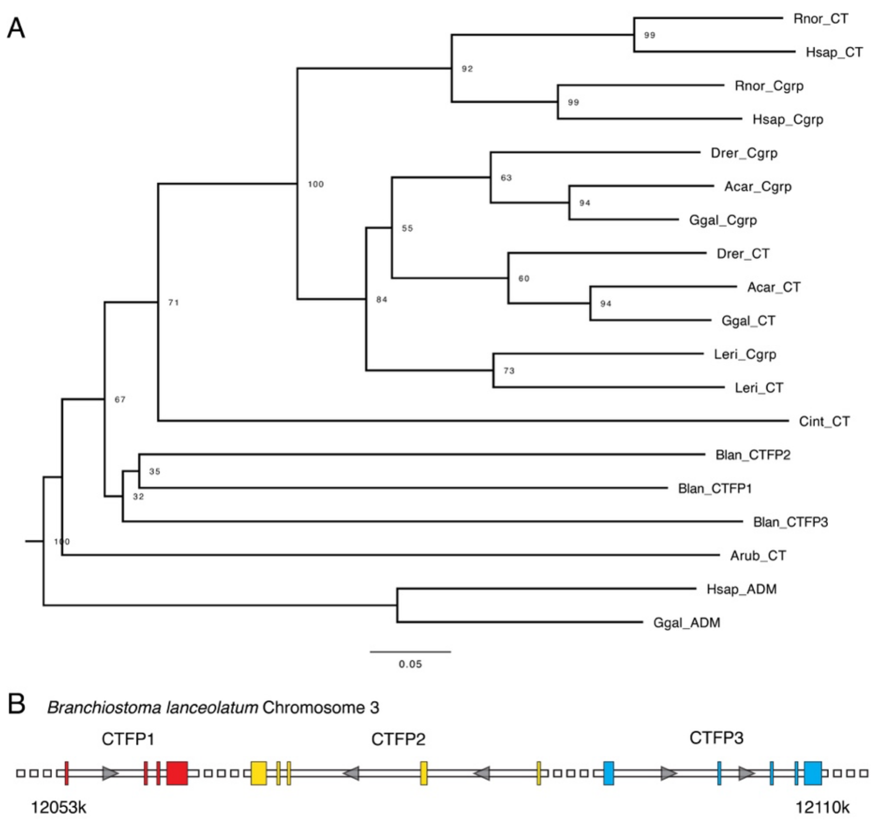

Fig. S4. Phylogeny of chordate calcitonin and calcitonin gene-related peptide sequences. a) Neighbour joining molecular phylogeny of chordate calcitonin (CT) and calcitonin gene-related peptide (Cgrp) sequences reveals a lineage-specific triplication of the locus encoding the calcitonin family protein (CTFP) in the amphioxus, *Branchiostoma lanceolatum*. Sequences used for this phylogenetic analysis are listed in Table S1. **b)** Genes encoding *B. lanceolatum* CTFP1-3 are arranged in a cluster on chromosome 3. *Acar, Anolis carolinensis; Arub, Asterias rubens; Blan, Branchiostoma lanceolatum; Cint, Ciona intestinalis; Drer, Danio rerio; Ggal, Gallus gallus; Hsap, Homo sapien; Leri, Leucoraja erinacea; Rnor, Rattus norvegicus*.

Development • Supplementary information

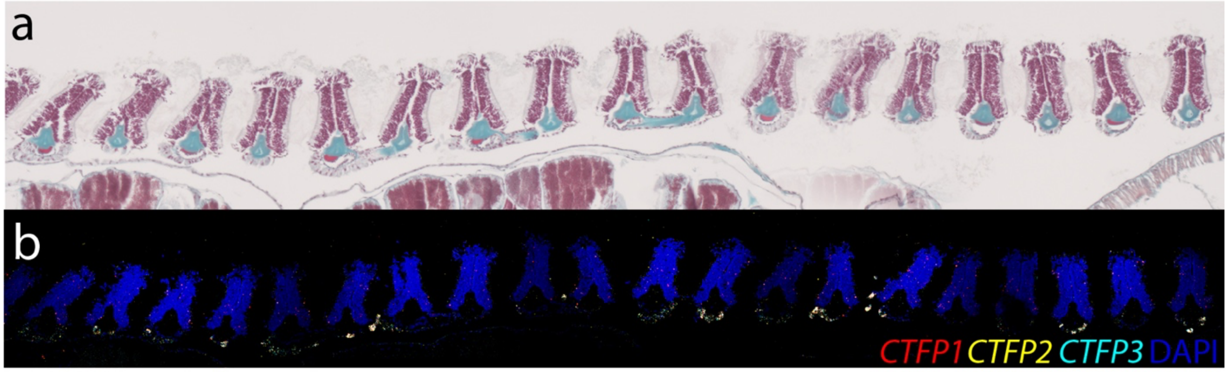

Fig. S5. CTFP expression in the pharyngeal arches of the amphioxus, *Branchiostoma lanceolatum*. a) Low-power view of the pharyngeal bars of an adult *Branchiostoma lanceolatum*, stained with Masson's Trichrome. This is a lower magnification image of the section featured in Fig. 5G of the main article. **b)** *CTFP1-3* are co-expressed in the atrial cells of each *B. lanceolatum* pharyngeal bar.
