# Supplementary material for: A pre-vertebrate endodermal origin of calcitonin-producing neuroendocrine cells
Source: Development. 2024 Aug 7;151(20):dev202821. doi: 10.1242/dev.202821 (PMC11698069; doi:10.1242/dev.202821)
Supplement: Supplementary file 1 [file develop-151-202821_review_history.pdf]

A pre-vertebrate endodermal origin of calcitonin-producing neuroendocrine cells

Jenaid M Rees, Katie Kirk, Giacomo Gattoni, Dorit Hockman, Victoria A Sleight, Dylan Ritter, Elia Benito-Gutierrez, Ela W Knapik, Gage Crump, Peter Fabian and Andrew Gillis
DOI: 10.1242/dev.202821

Editor: James Briscoe

Review timeline

Original submission:	22 February 2024
Editorial decision:	2 May 2024
First revision received:	20 June 2024
Accepted:	25 June 2024

Original submission

First decision letter

MS ID#: DEVELOP/2024/202821

MS TITLE: A pre-vertebrate endodermal origin of calcitonin-producing neuroendocrine cells

AUTHORS: Jenaid M Rees, Katie Kirk, Giacomo Gattoni, Dorit Hockman, Dylan Ritter, Elia Benito-Gutierrez, Ela W Knapik, Gage Crump, Peter Fabian, and Andrew Gillis

I have now received all the referees' reports on the above manuscript, and have reached a decision. The referees' comments are appended below, or you can access them online: please go to BenchPress and click on the 'Manuscripts with Decisions' queue in the Author Area.

The referees express interest in your work, but have some significant criticisms and recommend a substantial revision of your manuscript before we can consider publication. As you will see, there is a difference of opinion amongst the referees. While two referees found the evidence provided by the authors convincing, Reviewer 1 is not convinced and raises contradicting studies that have come to a different conclusion. On balance, my view is that it is important that your data are available for the community to assess, however, you should improve and extend the discussion of how your findings can be reconciled with the previous work cited by Reviewer 1. In addition, please attend to the other issues raised by the referees.

If you are able to revise the manuscript along the lines suggested, which may involve further experiments, I will be happy receive a revised version of the manuscript. Your revised paper will be re-reviewed by one or more of the original referees, and acceptance of your manuscript will depend on your addressing satisfactorily the reviewers' major concerns. Please also note that Development will normally permit only one round of major revision. If it would be helpful, you are welcome to contact us to discuss your revision in greater detail. Please send us a point-by-point response indicating your plans for addressing the referees' comments, and we will look over this and provide further guidance.

Please attend to all of the reviewers' comments and ensure that you clearly highlight all changes made in the revised manuscript. Please avoid using 'Tracked changes' in Word files as these are lost in PDF conversion. I should be grateful if you would also provide a point-by-point response detailing how you have dealt with the points raised by the reviewers in the 'Response to Reviewers' box. If you do not agree with any of their criticisms or suggestions please explain clearly why this is so.

© 2024. Published by The Company of Biologists under the terms of the Creative Commons Attribution License (https://creativecommons.org/licenses/by/4.0/).

Reviewer 1

Advance summary and potential significance to field

1 Localization of Ci-CT mRNA in the gills of the ascidian Ciona intestinalis is already shown by in situ hybridization (FEBS J 276:4437-4447, 2009).
Localizations of mRNAs for three CT/CGRP family peptides (Bf-CTFP1, Bf-CTFP2 and Bf-CTFR3) are shown in the amphioxus by in situ hybridization (J Biol Chem 291:2345-2356, 2016).

2 Many papers concerning the morphology of fish ultimobranchial glands including zebrafish and skate, have been published by using immunohistochemistry, in situ hybridization and electron microscopy. Since the fish ultimobranchial cells exhibit characteristics of epithelial cells and often form follicle structures, nobody considers that the fish ultimobranchial glands have a relationship with neural crest cells.
Zebrafish tfap2amob;foxd3mos mutants lack neural crest cells. The authors exhibit that ultimobranchial cells are still observed in the mutants.
Sox10>dsRed line labels all neural crest cells with DsRed. The authors indicate no DsRed cells in zebrafish.

Comments for the author

Dr. Gillis and coworkers have insisted in the manuscript that calcitonin-producing C cells are derivatives of the endodermal origin in all animal species.

Main comments

1. The chicken ultimobranchial gland is unique in several phylogenic aspects; the organ is located between the vagus, especially distal vagal ganglion, and the recurrent laryngeal nerves. Furthermore, from E8.0 the carotid body is always located cranially close to the ultimobranchial gland.
Since the carotid body is supplied by many nerve fibers from both the vagus and recurrent laryngeal nerves, the ultimobranchial gland also receives numerous fibers derived from both nerves (Am J Anat 182:353-368, 1988).
2. Calcitonin-immunoreactive C cells are dispersed in the large nerve bundles in the chicken ultimobranchial gland (Anat Rec 224:43-54, 1989). The cells are also distributed in the distal vagal ganglion and the recurrent laryngeal nerve located close to the gland. The property moving within nerve fibers is specific for neural crest cells but not for endodermal cells.
3. The avian ultimobranchial C cells are derivatives of neural crest cells.
This is a generally accepted opinion. It is necessary to indicate the enough supporting evidence to change the accepted opinion. Many manuscripts concerning the development of chick ultimobranchial body have been published. The neural crest cells immunoreactive for TuJ1, which are derived from the distal vagal ganglion, begin to enter the chick ultimobranchial body at E5.0 (J Comp Neurol 359:1-14, 1995). At E6.0, the ultimobranchial body is located between the distal vagal ganglion and enteric ganglia.
Many neuronal cells immunoreactive for TuJ1 and PGP9.5, derived from the both ganglia, surround the ultimobranchial body. The invasion of nerve cells and neuronal fibers into the ultimobranchial body continues until E8.0.
4. At E10 when the authors first examined the ultimobranchial body, the organ is already matured. At this time, the neural crest cells are completely differentiated into C cells.
5. The C cells of chick ultimobranchial gland exhibit the characteristics different from the cells of other animal species. The chick C cells are immunoreactive for tyrosine hydroxylase and enkephalin (Brain Res 852:453-462 2000; Cell Tissue Res 274:257-265, 1993; J Comp Neurol 304:373-386, 1991).
Electron microscopic study indicates that the C cells frequently protrude a long cytoplasmic process and axon terminals are directly apposed on the C cells (Anat Embryol 188:561-570, 1993). Schwann cell perikaryon enclosing numerous axons comes into close contact with the C cells. Furthermore, the chick ultimobranchial C cells retain neuronal properties for long term. When the C cells

© 2024. Published by The Company of Biologists under the terms of the Creative Commons Attribution License (https://creativecommons.org/licenses/by/4.0/).

obtained from chick ultimobranchial body at E13.0 were cultured for 7 days, the cells protruded a very long neurites reaching 300 μm (Brain Res 905:1-11, 2001). The C cells cultured in the presence of nicotinamide facilitated the manifestation of neuronal features (J Comp Neurol 492:334-349, 2005).

Thus, all manuscripts published indicate the neural crest origin of chick C cells.

Minor comment

Introduction, paragraph 1; add the reactions of C cells to hypercalcemia and hypocalcemia.
C cells react sensitively to increased serum calcium level. In particular the canine thyroid C cells reveal increased mitotic activity and form very large cell groups after hypercalcemia induced by chronic treatment of $CaCl_2$ and Vitamin D3 (Dev Dyn 245:323-341, 2016). Furthermore, the C cells become hypertrophic and display a conspicuous degranulation. Hypocalcemic tetany is induced in the dogs after parathyroidectomy. The C cells are filled with enlarged secretory granules under the hypocalcemic condition.

Reviewer 2

Advance summary and potential significance to field

Using transplantation, genetic lineage tracing or, alternatively, direct cellular labeling, this paper shows that foregut/pharyngeal endoderm constitute the embryonic origin of calcitonin-producing C-cells in birds, fishes and some protochordate species. The results confirm previous similar findings in mouse, and should once and for all dismiss the erroneous concept, still prevailing in many textbooks, of a neural crest origin of this neuroendocrine lineage.

C-cell development is an endodermal trait throughout the animal kingdom.

Comments for the author

Most if not all results are based on imaging documented by representative images, which is fine. However, although numbers of examined animals are indictated in the text, it would be good if this is shown graphically or in a table with the appropriate statistical analysis, to corroborate qualitative data.

In Fig. 2aa´and b, there seem to be two cell populations, the ultimobranchial body (Ub, encircled) and additional cells surrounding the Ub. Please clarify the identity of the second cell type, and whether those cells are Calcitonin+ or not. Based on Fig. 2b, it is not possible to distinguish if both are included.

Regarding dye labeling of pharyngeal endoderm (derivatives) through uptake of DiI in skate, the (unlikely) possibility that neural crest cells might already be integrated within the endodermal epithelium should be mentioned and shortly discussed. Notably, lineage tracing of Wnt1+ NCC ruled out this possibility in mouse embryos (Johansson et al, Development 2015).

Reviewer 3

Advance summary and potential significance to field

Using fatemapping techniques and immunostaining or hybridization chain reaction (HCR) this paper clarifies the embryonic origin of Calcitonin-producing cells in vertebrates. These cells have previously been suggested to arise from endoderm in mammals but from the neural crest in birds. Grafting GFP-labeled chick donor cells into unlabelled hosts, the authors now show that these cells are also endoderm- and not neural crest-derived in chick embryos. They further use genetic lineage labelling in zebrafish and vital dye lineage tracing in a skate to demonstrate that this endodermal origin is the ancestral vertebrate pattern.

© 2024. Published by The Company of Biologists under the terms of the Creative Commons Attribution License (https://creativecommons.org/licenses/by/4.0/).

Using HCR for calcitonin genes in Ciona and amphioxus They further show that endodermal calcitonin producing cell predated the origin of vertebrates (and thus, the neural crest).

The study nicely resolves conflicting previous reports about the origin of Calcitonin producing cells in vertebrates and convincingly demonstrates their endodermal provenance. It furthermore provides new information on the phylogenetic distribution of these cells, suggesting that they were already present in non-vertebrate chordates. The paper is well written and well documented and makes an important contribution to our understanding of basic vertebrate embryology.

Comments for the author

I have no suggestions for changes.

First revision

Author response to reviewers' comments

18 June 2024

Dear James,

Thank you very much for reviewing our manuscript entitled *“**A pre-vertebrate endodermal origin of calcitonin-producing neuroendocrine cells**”* (DEVELOP/2024/202821) for *Development*. Thank you, also, to our three reviewers for their constructive feedback on the manuscript. Below, I have included a point-by-point list of the changes that we have made to our manuscript in response to reviewer feedback. All changes to manuscript text are highlighted in red (here, and in the main manuscript file).

Reviewer 1

Localization of Ci-CT mRNA in the gills of the ascidian *Ciona intestinalis* is already shown by *in situ*
hybridization (FEBS J 276:4437-4447, 2009).

We do cite this report in our manuscript, but our findings expand on those reported in the paper by Sekiguchi et al. (2009). Their previous ISH experiments were performed in wholemount, and so only resolved expression of *Ci-CT* to the general area of the pharynx. Our HCR ISH on paraffin sections resolved this expression further, down to a histologically distinct cell type within the papillae of the pharyngeal lining. To reflect this, we have revised the text as follows:

C. intestinalis has a single calcitonin-like gene (Ci-CT), and expression of this gene has been previously reported in the C. intestinalis pharynx by wholemount mRNA in situ hybridization (Sekiguchi et al., 2009; Hamada et al., 2011; Sataka and Sasakura, 2023). To expand on previous reports, we tested for the expression of Ci-CT in paraffin sections of the entire adult C. intestinalis using mRNA in situ hybridization by chain reaction (HCR).

Localizations of mRNAs for three CT/CGRP family peptides (Bf-CTFP1, Bf-CTFP2 and Bf-CTFR3) are shown in the amphioxus by in situ hybridization (J Biol Chem 291:2345-2356, 2016).

We also cite this previous report in our manuscript. In this report, expression of Bf-CTFP1-3 are reported in the head (likely brain) region and in the digestive tract of *B. floridae*. However, there are no expression data for the pharynx, which is the focus of our analysis (given the homology of amphioxus and vertebrate pharyngeal endodermal pouches, and the pharyngeal endodermal pouch origin of the ultimobranchial gland in vertebrates). Nevertheless, we have now expanded our reference to this previous work to include mentions of the expression in the brain and gut as follow:

Cephalochordates have three calcitonin genes encoding CTFPs (calcitonin family proteins), and

© 2024. Published by The Company of Biologists under the terms of the Creative Commons Attribution License (https://creativecommons.org/licenses/by/4.0/).

these three genes are expressed in different combinations in neurons of the amphioxus larva and adult, and in isolated cells within the adult midgut and hindgut (Sekiguchi et al., 2016; Sekiguchi, 2018; Gattoni et al., 2023).

As a major point of revision, Reviewer 1 has listed several additional indirect pieces of evidence that have been taken in support of a neural/neural crest origin of C-cells in chick. These include unique patterns of C-cell innervation, C-cells with some neural-like morphological features, C-cells exhibiting neural marker gene expression, and histological evidence of C-cells residing within nerve bundles and an adjacent ganglion. We originally limited our discussion to other cell lineage tracing experiments, but we thank the Reviewer for raising these concerns, as it has led to us significantly expanding our Discussion to include these (and other) additional studies. We believe that this has really improved the strength of our paper.

We are very aware that, as the reviewer notes, current opinion is that avian C-cells derive from the neural crest – indeed, it is for this reason that our findings of a very different embryonic origin of C-cells in chick serves as an important new contribution to the field. The reviewer suggests that because C-cells in chick exhibit neural-like characteristics, they must be neuroectoderm/neural crest-derived. In our revised discussion, we make the point that, while circumstantial evidence has contributed to a widespread acceptance of a neural crest origin of avian C-cells, only cell lineage tracing experiments can *directly* resolve embryonic germ layer origin. We also cite recent studies that challenge long-held assumptions about the diversity of endodermal derivatives, including examples of endoderm giving rise to cell types previously thought to derive exclusively from ectoderm. Finally, we discuss how the unique features of avian C-cells may be re-interpreted considering their endodermal origin. Revised text and new citations are as follows:

Discussion

Conservation of C-cell embryonic origin within jawed vertebrates

Avian C cells exhibit some striking and unique neural-like features that are not shared with those of other vertebrate taxa. For example, chick C cells may be directly innervated by nerve fibres originating from the vagus and recurrent laryngeal nerves (Kameda et al., 1988), unlike mammalian C cells, which receive no direct innervation. Chick C cells are also distinct from the C cells of other vertebrates in their co-expression of the neural markers tyrosine hydroxylase and enkephalin, and in their possession of elongated cell processes that contact those (or somata) of adjacent C cells during earlier stages of their differentiation (Kameda, 1991; Kameda et al., 1993). The discovery of calcitonin-positive C cells within the nerve bundles that invade the UB and the adjacent nodose ganglion (Kameda, 1989), along with evidence of a neural crest origin from previous lineage tracing experiments (Le Douarin and Le Lièvre, 1970; Pearse and Polak, 1971; Polak et al., 1974), led to a model in which neural crest progenitor cells traveling from the nodose ganglion and the vagus nerve surround and invade the UB before differentiating into C cells by E10 (Kameda, 1995). Importantly, the epithelial cells that line the cystic cavities of the post-hatching and adult chicken UB also stain positively for calcitonin (Kameda, 1984). Based on the assumption that the latter derive from the endodermal epithelium of the UB, it has been suggested that avian C cells may have a dual embryonic origin from neural crest and endoderm (Kameda, 1995; 2017), with neural crest-derived C cells possibly representing an avian innovation.

While histological snapshots can provide invaluable data on the progression of differentiation and marker expression within tissues through development, they cannot determine cell lineage. Our study finds that chick C-cells do not derive from the neural crest, and that previous lineage tracing experiments using quail-chick chimeras likely recovered a neural crest contribution to connective tissue or other cell types within the parenchyma of the UB, rather than to the calcitonin-expressing C-cells. The challenges of sequential imaging of cell type markers and cell lineage (quail vs. chick), using formaldehyde-induced fluorescence/immunohistochemistry and Feulgen staining, respectively, could account for earlier interpretations of UB C-cells as a derivative of the neural crest. We performed cell lineage tracing experiments by grafting neural folds from GFP+ chick embryos into wild-type hosts, and unlike earlier experiments, this approach allowed us to image cell lineage and cell type marker expression simultaneously. We found no evidence of a neural crest contribution to calcitonin-expressing C-cells, but rather found by direct labeling of pharyngeal endoderm with the lipophilic dye CM-DiI that this tissue gives rise to the

© 2024. Published by The Company of Biologists under the terms of the Creative Commons Attribution License (https://creativecommons.org/licenses/by/4.0/).

calcitonin-expressing C-cells of the UB at E10. Immuno-electron-microscopical studies of the chick UB reported three types of neuroendocrine cells (types-I, II and III), based on differences in the size, nature, and contents of their secretory granules (Kameda et al., 1993). Immunolabelling using the protein A-colloidal gold method revealed that calcitonin co-localised with the secretory granules of types-I and -II cells, while enkephalin co-localised with the granules of types-II and -III cells. It was noted that the type- III neuroendocrine cells (which express enkaphalin, but not calcitonin) resemble C cells in an earlier state of differentiation (Kameda, 1991; Kameda et al., 1993). Rather than representing a C- cell precursor, we suggest instead that these cells could represent an additional, avian-specific neuroendocrine cell type, distinct from the endoderm-derived calcitonin-expressing C-cells, and that this cell type could derive from neural ectoderm or the neural crest.

The endodermally-derived C-cells in mammals have a well-documented propensity for migration, despite their embryonic origin within an epithelium. As discussed above, mammalian C-cells develop from cells of the transient UB, eventually invading and dispersing within the developing thyroid primordium. Additionally, in cat, rabbit, goat and dog, C-cells may be found not only in the thyroid gland, but also in varying numbers within the neighbouring parathyroid and thymus glands (Kameda, 1971; 1981). It is therefore plausible that calcitonin-expressing C-cells within the avian vagus nerve and nodose ganglion could reflect the migration of C-cells into, rather than an origin from, those neural tissues. Finally, it has become increasingly evident that endoderm has the capacity to differentiate into cell types long thought to derive exclusively from other embryonic germs layers. Endoderm can give rise to tooth ameloblasts (Soukup et al., 2008), neuroendocrine cells of the anterior pituitary (Fabian et al., 2020) and even neurons (Wei et al., 2011; Nakanishi et al., 2012) – all cell types that are widely regarded as ectodermal derivatives. These observations stress the need for cell lineage tracing, in addition to data on cell phenotypes (e.g., morphology and gene expression), to confidently infer a cell type's germ layer origin.

Prior to this study, no data were available on the embryonic origin of vertebrate C-cells outside of tetrapods, though an endodermal origin has been assumed (Kameda, 2017). In zebrafish, the UB consists of several small epithelial follicles, and sits beneath the circular musculature of the esophagus just rostral to the sinus venosus (Yamane, 1978), whereas in batoid elasmobranch fishes (skates and rays), the UB forms initially as a single large follicle but subsequently takes the form of a solid mass of cells (Kameda, 2017, and this study). Both teleost and batoid UBs express calcitonin mRNA and/or protein (McMillan et al., 1976; Takei et al., 1991; Hidaka and Suzuki, 2004; Alt et al., 2006). Here, we formally demonstrate, by lineage tracing, that the UB C-cells of zebrafish and skate both derive from endoderm, with no evidence of a neural crest contribution. Alongside lineage tracing data from mouse (Kameda et al., 2007; Johansson et al., 2015) and chick (this study), these findings point to a broadly conserved endodermal origin of C- cells across jawed vertebrates (tetrapods, bony fishes, and cartilaginous fishes).

Introduction, paragraph 1; add the reactions of C cells to hypercalcemia and hypocalcemia. C cells react sensitively to increased serum calcium level. In particular, the canine thyroid C cells reveal increased mitotic activity and form very large cell groups after hypercalcemia induced by chronic treatment of CaCl2 and Vitamin D3 (Dev Dyn 245:323-341, 2016). Furthermore, the C cells become hypertrophic and display a conspicuous degranulation. Hypocalcemic tetany is induced in the dogs after parathyroidectomy. The C cells are filled with enlarged secretory granules under the hypocalcemic condition.

This very specific feature of the cellular changes in canine C-cells under hyper- and hypocalcemia doesn't fit easily into our first paragraph, which focuses more on the general function of C-cells. We would prefer to keep the current version of our first paragraph.

Reviewer 2

Most if not all results are based on imaging documented by representative images, which is fine. However, although numbers of examined animals are indictated in the text, it would be good if this is shown graphically or in a table with the appropriate statistical analysis, to corroborate qualitative data.

© 2024. Published by The Company of Biologists under the terms of the Creative Commons Attribution License (https://creativecommons.org/licenses/by/4.0/).

Because of the relatively small samples sizes and the binary nature of our findings (e.g., no NC-derived C-cells observed in Sox10>dsRed zebrafish, and completely endodermally-derived C-cells in sox17>mCherry zebrafish), there aren't really statistical tests that we can perform on these data. Also, with our skate lineage tracing, the CM-DiI label dilutes over time, so counting CM-DiI+ cells is also not particularly effective (hence our scoring of animals as either having or not having evidence of a lineage contribution to the C-cells of the UB). But we do agree with the reviewer that have a tabular summary of our various lineage tracing experiments would be helpful, and we have added this as a new Table 1.

In Fig. 2aa´and b, there seem to be two cell populations, the ultimobranchial body (Ub, encircled) and additional cells surrounding the UB. Please clarify the identity of the second cell type, and whether those cells are Calcitonin+ or not. Based on Fig. 2b, it is not possible to distinguish if both are included.

Based on morphology, we suspect that this is a nerve that either contains some calcitonin-expressing C- cells, or that is being stained by our anti-calcitonin antibody due to its cross-reacticity with CGRP (which is expressed in many neuronal subtypes). We have added the following statement to address this in the manuscript:

In Fig. 1e, apparent calcitonin expression is also present in a nerve adjacent to the UB. This staining could reflect calcitonin-expressing C cells within the nerve (see Discussion), or cross-reaction of our anti-calcitonin antibody with calcitonin gene-related peptide in the nerve.

Regarding dye labeling of pharyngeal endoderm (derivatives) through uptake of DiI in skate, the (unlikely) possibility that neural crest cells might already be integrated within the endodermal epithelium should be mentioned and shortly discussed. Notably, lineage tracing of Wnt1+ NCC ruled out this possibility in mouse embryos (Johansson et al, Development 2015).

This is an excellent point. Our pharyngeal endodermal labeling experiments are performed at embryonic stage (S)18, and at this stage, neural crest cells are specified within the dorsal neural tube but have not yet started to migrate. It is therefore impossible that our endodermal CM-DiI labeling has contaminated post-migratory neural crest cells that have already invaded the endodermal epithelium. To clarify this in the text, we have added the following statement (and an additional citation which shows the timing of neural crest cell specification and migration in the little skate):

Most importantly, at the stage in which we conduct our pharyngeal endodermal labeling experiments, pre- migratory neural crest cells are specified in the dorsal neural tube, but have not yet begun to migrate (Gillis et al., 2017), thus precluding the possibility of inadvertent neural crest cell contamination with our CM-DiI labelling.

Reviewer 3

Reviewer 3 had no recommendations for changes.

Additional changes

In addition to the revisions recommended by the reviewers, we have also revised the manuscript to conform with *Development* formatting as follows:

- We have converted citations from numbered to named.
- We have broken our combined Results and Discussion section into separate Results and Discussion sections.
- We have reformatted our reference list according to the *Development* style.

Thank you for reconsidering our revised submission,

© 2024. Published by The Company of Biologists under the terms of the Creative Commons Attribution License (https://creativecommons.org/licenses/by/4.0/).

Andrew Gillis, Ph.D.

Second decision letter

MS ID#: DEVELOP/2024/202821

MS TITLE: A pre-vertebrate endodermal origin of calcitonin-producing neuroendocrine cells

AUTHORS: Jenaid M Rees, Katie Kirk, Giacomo Gattoni, Dorit Hockman, Victoria A Sleight, Dylan Ritter, Elia Benito-Gutierrez, Ela W Knapik, Gage Crump, Peter Fabian, and Andrew Gillis
ARTICLE TYPE: Research Article

I am happy to tell you that your manuscript has been accepted for publication in Development, pending our standard publication integrity checks.

Reviewer 2

Advance summary and potential significance to field

I´m entirely happy with the manuscript revision adhering to the major points of criticism, which in my mind were few.

I also fully agree with the revised, extended version of Discussion on the seemingly controversial issue of avian C-cell origin, which provides alternative explanations to previous misleading findings suggestive of a neuroectodermal/neural crest derivation.

This paper makes important conclusions which should once and for all let the neural crest concept or rather hypothesis be buried in favor of the endoderm origin of neuroendocrine C-cells across species. Thank you.

Comments for the author

I´m entirely happy with the manuscript revision adhering to the major points of criticism, which in my mind were few.

I also fully agree with the revised, extended version of Discussion on the seemingly controversial issue of avian C-cell origin, which provides alternative explanations to previous misleading findings suggestive of a neuroectodermal/neural crest derivation.

This paper makes important conclusions which should once and for all let the neural crest concept or rather hypothesis be buried in favor of the endoderm origin of neuroendocrine C-cells across species. Thank you.

Reviewer 3

Advance summary and potential significance to field

All comments of previous reviews have been addressed satisfactorily

Comments for the author

All comments of previous reviews have been addressed satisfactorily

© 2024. Published by The Company of Biologists under the terms of the Creative Commons Attribution License (https://creativecommons.org/licenses/by/4.0/).
